# Development of Flexible Plasticized Ion Conducting Polymer Blend Electrolytes Based on Polyvinyl Alcohol (PVA): Chitosan (CS) with High Ion Transport Parameters Close to Gel Based Electrolytes

**DOI:** 10.3390/gels8030153

**Published:** 2022-03-02

**Authors:** Niyaz M. Sadiq, Shujahadeen B. Aziz, Mohd F. Z. Kadir

**Affiliations:** 1 Hameed Majid Advanced Polymeric Materials Research Lab., Physics Department, College of Science, University of Sulaimani, Qlyasan Street, Sulaimani 46001, Iraq; niyaz.sadiq@univsul.edu.iq; 2 Department of Civil Engineering, College of Engineering, Komar University of Science and Technology, Sulaimani 46001, Iraq; 3 Centre for Foundation Studies in Science, University of Malaya, Kuala Lumpur 50603, Malaysia; mfzkadir@um.edu.my

**Keywords:** polymer blend electrolytes, NaBr salt, glycerol plasticizer, XRD and FTIR methods, circuit design, ion transport parameters, dielectric properties

## Abstract

In the current study, flexible films of polyvinyl alcohol (PVA): chitosan (CS) solid polymer blend electrolytes (PBEs) with high ion transport property close enough to gel based electrolytes were prepared with the aid of casting methodology. Glycerol (GL) as a plasticizer and sodium bromide (NaBr) as an ionic source provider are added to PBEs. The flexible films have been examined for their structural and electrical properties. The GL content changed the brittle and solid behavior of the films to a soft manner. X-ray diffraction (XRD) and Fourier transform infrared (FTIR) methods were used to examine the structural behavior of the electrolyte films. X-ray diffraction investigation revealed that the crystalline character of PVA:CS:NaBr declined with increasing GL concentration. The FTIR investigation hypothesized the interaction between polymer mix salt systems and added plasticizer. Infrared (FTIR) band shifts and fluctuations in intensity have been found. The ion transport characteristics such as mobility, carrier density, and diffusion were successfully calculated using the experimental impedance data that had been fitted with EEC components and dielectric parameters. CS:PVA at ambient temperature has the highest ionic conductivity of 3.8 × 10 S/cm for 35 wt.% of NaBr loaded with 55 wt.% of GL. The high ionic conductivity and improved transport properties revealed the suitableness of the films for energy storage device applications. The dielectric constant and dielectric loss were higher at lower frequencies. The relaxation nature of the samples was investigated using loss tangent and electric modulus plots. The peak detected in the spectra of tanδ and M” plots and the distribution of data points are asymmetric besides the peak positions. The movements of ions are not free from the polymer chain dynamics due to viscoelastic relaxation being dominant. The distorted arcs in the Argand plot have confirmed the viscoelastic relaxation in all the prepared films.

## 1. Introduction

Renewable energy sources are those derived from naturally replenishing sources, including the sun, wind, storms, seas, seeds, algae, geothermal, and biodegradable polymer materials, and they have attracted great interest due to the growing oil crisis and environmental concerns [1,2]. Polymer electrolyte (PE) science encompasses a wide range of disciplines such as polymer science, organic chemistry, electrochemistry, and inorganic chemistry [3]. Polymers are a prominent issue in material science lately, particularly solid state solutions, which are an example of ion conducting polymers [4]. Because they are important in energy storage devices, including fuel cells, hybrid power sources, supercapacitors, and batteries, solid PEs (SPEs) have been attracting a lot of recent support [5,6,7,8]. Polar polymers can coordinate with the cations in the salt, causing it to dissolve. This is due to the presence of functional groups with high electronegativity [9]. To keep pace with rapid technological change, a new generation of highly efficient energy sources must be developed. Because of its use as an electrolyte, polymer-based ion conducting materials have sparked large interest in lithium batteries. Super-ionic conductors are solid substances in which charged atoms, known as ions, carry electric current [10]. These materials’ electrical conductivity is vastly different from that of conventional semiconductors. This is because the conductivity of semiconductors is determined by the mobility of light electrons. The conductivity of super-ionic conductors, on the other hand, is the result of ion mobility. Ions have a significant amount of mass and volume [11]. As a result, electric charge transport is linked to mass transfer in super-ionic conductors [10,11].

Chitin is the world’s second most abundant biopolymer, derived from fungus and insect cell walls, as well as crustacean exoskeletons [12]. Chitosan (CS), a biopolymer derived from chitin and used in a variety of medicinal and electrochemical devices, is safe, nontoxic, and biodegradable [13]. The backbone structure of CS differs from other biopolymers because it contains amino and hydroxyl functional groups [14]. Poly (vinyl alcohol) (PVA), on the other hand, is a water-soluble polymeric substance with high dielectric strength, strong charge storage capacity, and fascinating optical features [15]. In fact, the PVA molecule has a hydrophobic chain and a hydrophilic end group, the hydrophobic chains occupy space at the solid-liquid interface, while the hydrophilic end groups are extended in the outer aqueous phase. Thus, the steric hindrance and increase in the energy barrier would prevent the aggregation of the crystallinity [16]. The existence of polar groups with a high electron affinity in polar polymers is adequate to form coordination with the cation or surface groups of the fillers, resulting in a uniform nanocomposite [9,17]. PVA has a carbon chain backbone with hydroxyl groups attached, which can help build polymer composites by facilitating hydrogen bonding. Because of its high transparency and ability to form an oxygen barrier, this polymer is a suitable option for use in multilayer coatings for organic solar cells [18].

Ionic conductivity, dimensional stability, and mechanical stability are some of the latest economic and commercialization challenges in membrane technology research. Crystallinity and low ionic conductivity are two of the most significant drawbacks of SPEs [19]. Chemists and engineers are interested in the ionic conductivity of PEs since it is used in commercial electrochemical devices [20]. Both crystalline and amorphous phases exist in PEs. Polymers utilized as host materials in PEs are commonly semi-crystalline, despite the fact that ion transport occurs more frequently in amorphous rather than crystalline phases, as has long been recognized [21]. To overcome the drawbacks of SPEs and enhance conductivity, polymer blending and plasticizer addition are employed to improve ambient ionic conductivity. PVA and CS-based polymer blend complexes are simple to manufacture because of their well-controlled chemical and physical characteristics, such as toughness, homogeneity, and heat stability [22]. Blending CS and PVA is possible since they are both miscible [22]. A result of this interaction is that hydroxyl and amine groups in PVA and CS combine to form an ionic compound. For example, there are many previous works which enhance ionic conductivity by blending CS and PVA [22,23,24,25]. An XRD diffractogram revealed that the most amorphous blend host was a 50/50 mix of PVA and CS. Plasticizers may increase SPE’s DC electrical conductivity by dissociating PE ion aggregates and boosting the PE’s amorphous content [26].

GL, a colorless and odorless liquid, is widely available as an unavoidable by-product of the transesterification of vegetable oils used to make biodiesel, and it can also be obtained from more sustainable sources such as microalgae or cellulose. Because of its inexpensive cost, low toxicity, and unique physicochemical features, such as water solubility and hygroscopicity, it is commonly utilized in pharmaceutical formulations [27]. Increased biopolymer electrical conductivity may be achieved with the use of GL, a plasticizer rich in hydroxyl groups, which reduces the number of internal hydrogen connections between polymer chains. This opens up new applications, such as solid PE films. Electrochemical applications such as humidity sensors, rechargeable batteries, and fuel cells might benefit from this feature’s potential [28,29].

It is widespread to use dielectric relaxation spectroscopy to learn relaxation processes in complex systems from a basic perspective. Studying the dielectric characteristics of ion conducting polymers may help researchers understand more about ionic and molecular interactions. The nature of additives and temperature have a huge effect on the dielectric characteristics of ion conducting polymers [30]. The nature of charge transport in polymers has been investigated in order to better understand how these materials conduct electricity [31]. AC impedance spectroscopy is a method to look at the electrical and dielectric properties of materials. The goal of this research is to create a novel type of PE system based on a 50 percent PVA and 50 percent CS (1:1) blend that will act as a good polymer host. This study will look at the structural (XRD and FTIR), conductivity, and relaxation processes that make ions move. Particular attention is given to the exploration of the ion’s relaxation and movement in the PVA:CS-based ion conducting mix electrolyte membranes. Polymer films with high content of plasticizer will offer DC conductivities close sufficient to gel-like electrolytes. Because most polymer membranes with ions that are used in devices have a high conductivity, the system made in this study could be used as an electrolyte and separator for electrochemical device applications.

## 2. Results and Discussion

### 2.1. FTIR Study

**Figure 1:**
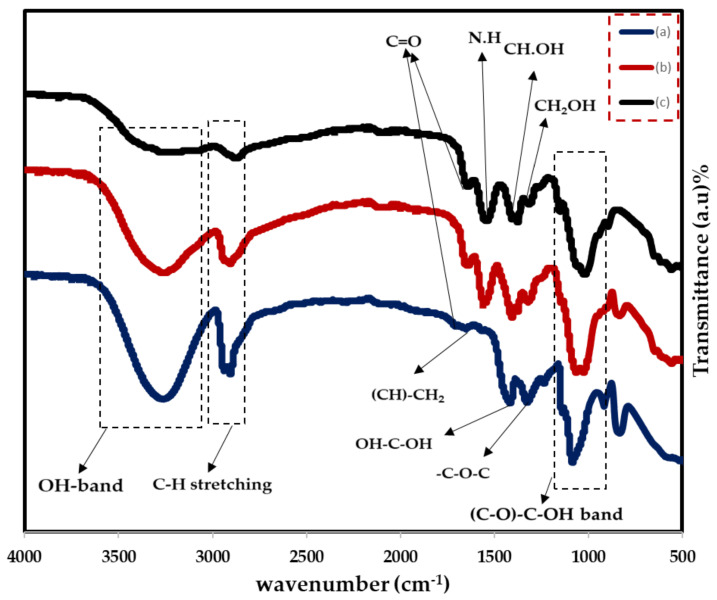
FTIR spectra for (**a**) pure PVA, (**b**) PVA:CS (0.5:0.5) blend, (**c**) pure CS.

The FTIR spectroscopy is used to investigate the chemical structure of the composite films and probable interactions between the functional groups of PVA and CS in PVA:CS polymer bleed films. Additionally, in this technique, the interaction of NaBr salt with the blended PVA:CS host polymer by modifying the location, intensity, and shape of the IR transmittance bands in the wavenumber range from 500 to 4000 cm^−1^ is explored. The vibrational peaks of OH, C–O, C–H, CH_2_, and C=O are used to identify the distinctive bands of PVA and CS polymers [32]. Blend polymer compatibility is shown by changes in the vibrational frequency of the peaks and improved amorphous phase in the PVA:CS system. Figure 1 shows the FTIR spectra of pure CS, PVA, and their blends. In Table 1, the FTIR peaks and their assignments are listed for pure polymers. Figure 1 shows that the OH bands in the PVA:CS system grow in size with decreased intensity, which indicates a reduction in PVA crystallinity. This is evident for the occurrence of complexation between the functional groups of the two polymers, which is clearly related to the OH band of the blend system [33]. The results of the XRD investigation are consistent with this discovery. By shifting the O–H peak position and intensity, the PVA hydrogen atoms and the CS oxygen atom are shown to establish hydrogen bonds with one other. Blending CS with PVA results in an expansion of the crystalline peak that corresponds to the C–O stretching mode and a decrease in its intensity [34]. It has been shown that adding CS to a mix diminishes the absorption band intensity at 834 cm^−1^, which corresponds to PVA’s C–C stretching.

**Figure 2:**
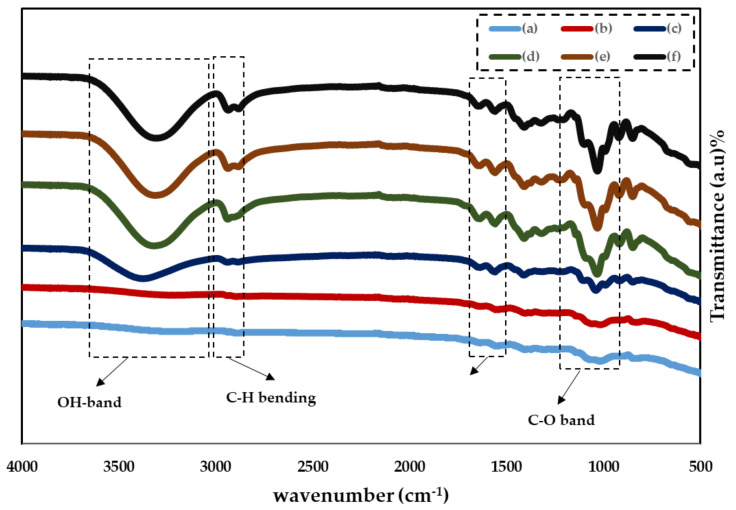
FTIR spectra of (**a**)CSPVNG0, (**b**) CSPVNG11, (**c**) CSPVNG22, (**d**) CSPVNG33, (**e**) CSPVNG44 and (**f**) CSPVNG55.

Figure 2 illustrates the FTIR spectra of various PVA:CS:NaBr:Gl  in the wavenumber range $500\text{–}4000{\;\mathrm{cm}}^{-1}$. [35] All samples show the key characteristic absorption peaks of CS, for instance the vibration of the amino group (NH_2_), O=C–NHR, and amine NH symmetric. The PVA structure has been connected to C–O plane bending, which commonly occurs at $1015\text{–}1031\;{\mathrm{cm}}^{-1}$. Furthermore, the peak at $1600\text{–}1700{\;\mathrm{cm}}^{-1}$ is due to C=O stretching of PVA’s acetate group, which is pushed to a lower wavenumber in doped samples [35].

There is motionless a noteworthy wide band at 3300–3500 cm^−1^, despite the possibility of overlap between the N–H and O–H stretching vibrations. Figure 2 shows that as the plasticizer is increased, the bands become more intense and the wavenumber decreases. In the bands of amine (NH2) and (O–H) groups, there is a trend towards lower wavenumbers, as seen in Table 2. This strongly suggests that complexation is happened between the electrolyte’s constituents [23,36]. The shift and reduction in relative strength of these bands is due to the electrostatic interaction between the ions and the functional groups of the CS:PVA polymer blend [37]. Furthermore, the N–H stretching vibration’s shift to lower wavenumbers shows that CS’s intermolecular and intramolecular hydrogen bonds have decreased [38]. Recent research has shown that the carboxyl (–C=O), hydroxyl (–OH), and amine (–NH) groups all have a role in salt interaction [39]. This band’s shifting and changing strength specify a superior interaction between the electrolyte’s blended host polymer, salt, and plasticizer. The vibrational and stretching modes in the FTIR spectra vary as a consequence of the interaction between the electrolyte components (shown in the table below) [22,40]. This enhanced interaction promotes ion dissociation, which is beneficial to the electrolytes’ ionic conductivity [40,41]. The rise in ionic conductivity is corroborated by this increase in intensity (see Figure 2).

### 2.2. XRD Study

**Figure 3:**
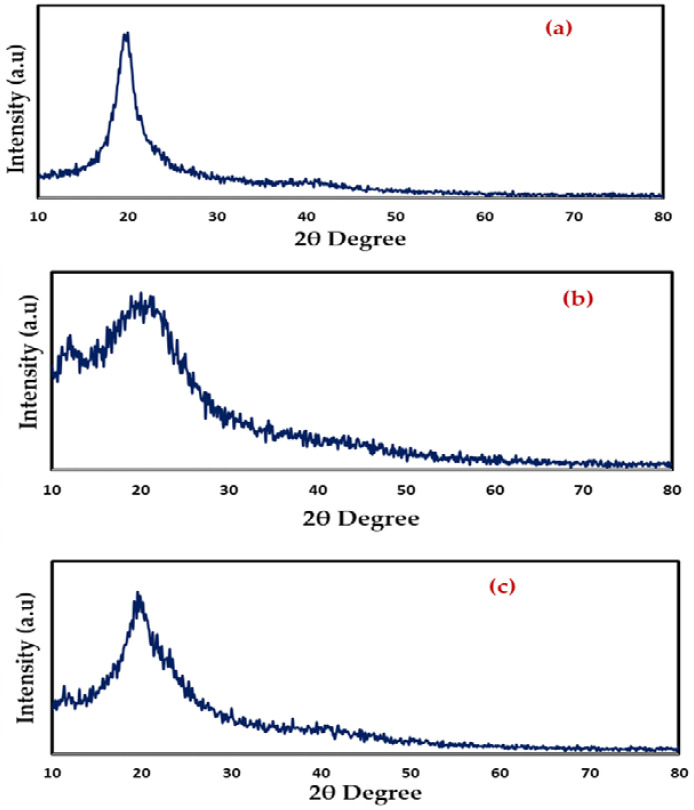
XRD pattern of (**a**) pure PVA film, (**b**) pure CS and (**c**) PVA: CS blend (50:50) films.

Figure 3 shows XRD results for room-temperature PVA, CS, and CS:PVA (a–c). In contrast to the semi-crystalline character of pure PVA (Figure 3a), the CS exhibits crystallinity maxima at some 2θdegree values (Figure 3b) [42]. Since OH groups are present everywhere throughout PVA and CS’s main chain, it is able to form strong inter- and intramolecular hydrogen bonds. Amorphous phases in PVA are responsible for the large peak at 2θ = 40.7° which is due to existing high water content [43]. The XRD pattern of CS: PVA (Figure 3c) revealed two hallows and smaller crystalline peaks in the current study. It is worth noting that, as the vast hallows demonstrate, CS: PVA is less crystalline than pure PVA or CS alone, and its structure is practically amorphous [44,45]. According to previous research, the amorphous structure of PEs is linked to large diffraction peaks [46].

**Figure 4:**
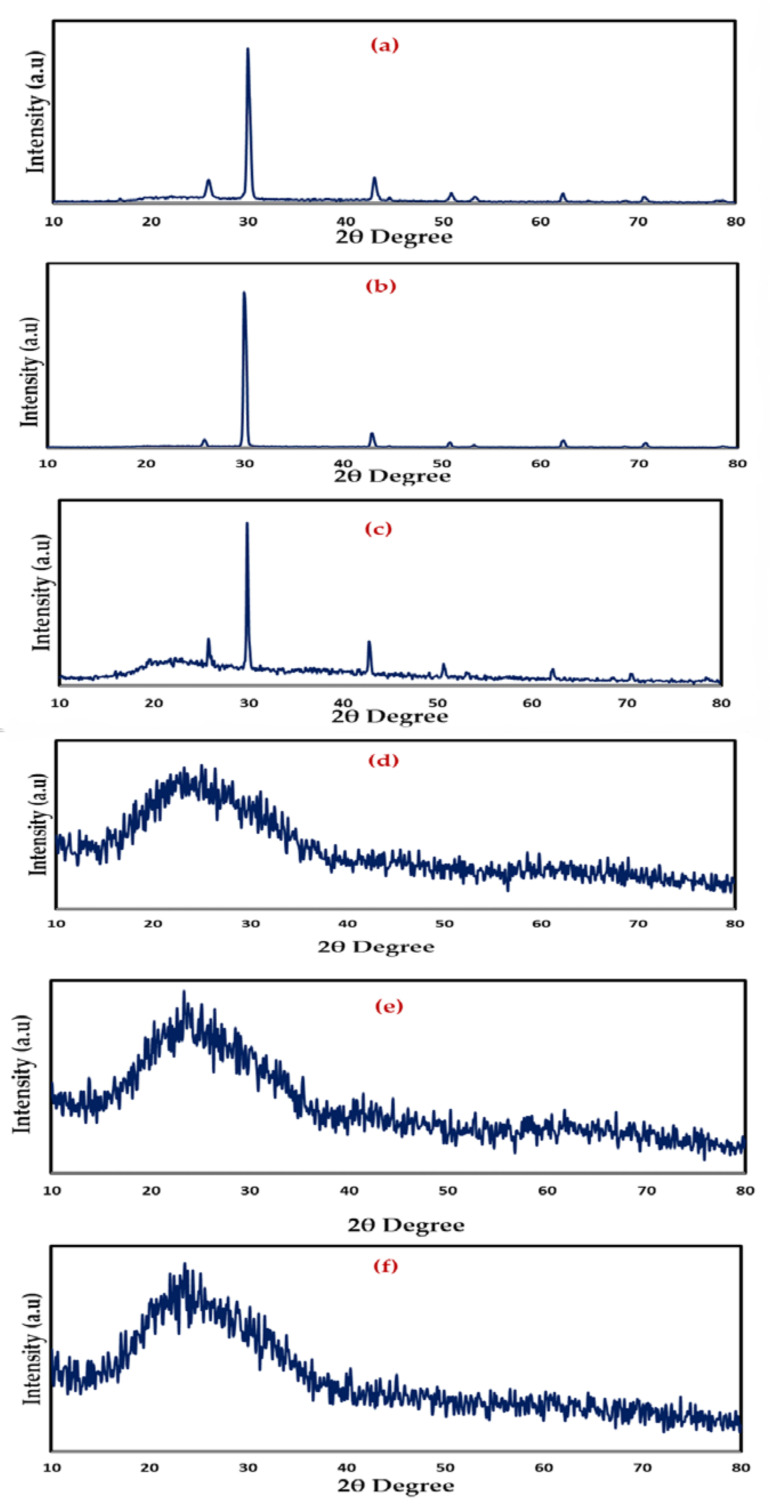
XRD Pattern of (**a**) CSPVNG0, (**b**) CSPVNG11, (**c**) CSPVNG22, (**d**) CSPVNG33, (**e**) CSPVNG44 and (**f**) CSPVNG55.

**Figure 5:**
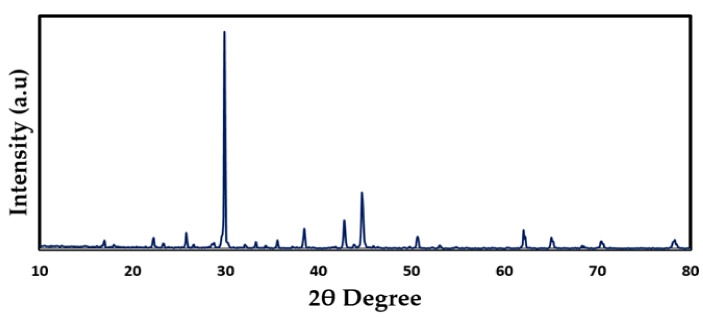
XRD pattern of pure NaBr.

**Figure 6:**
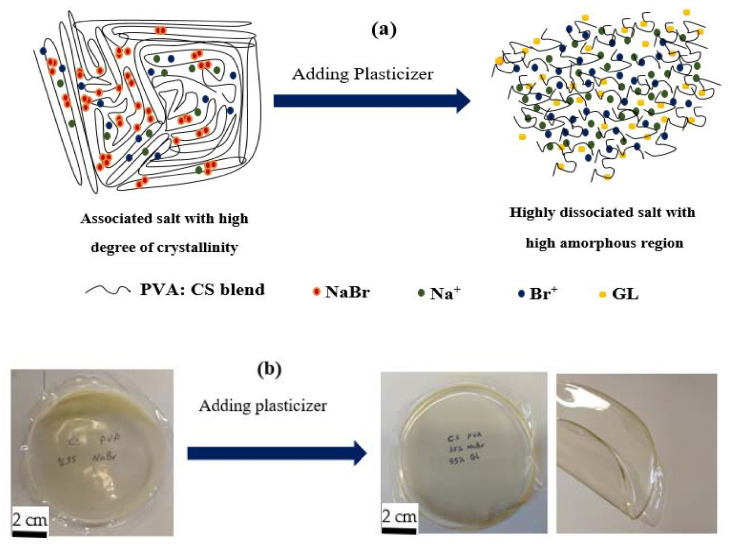
Role of plasticizer on (**a**) the ion dissociation and increase of flexibility and amorphous phase, and (**b**) the film with high content of plasticizer can easily be bending without deformation or tear. The last photograph shows the ability of the film to bending.

Meanwhile, when GL was added to these samples, the strength of the CS:PVA:NaBr peaks decreased, and the wide nature of the peaks improved, as seen in Figure 4a–f. These findings support the hypothesis that the PE has an amorphous structure that improves conductivity by increasing ionic diffusivity. Furthermore, the NaBr salt dissociates completely in the PE, leaving no peak associated with pure NaBr, as illustrated in Figure 5. The absence of hydrogen bonds between polymer chains is a likely cause for the intensity drop and broadening, indicating the presence of the amorphous phase in the samples [47]. Plasticized PEs are a type of PE made by adding low molecular weight chemicals to the polymer host, such as ethylene carbonate, propylene carbonate, and poly ethylene glycol (PEG) [48]. It is possible for plasticizers to reduce the number of active centers in polymer chains, hence decreasing the interactions between and within molecules [49]. Consequently, the three-dimensional structure generated during drying loses stiffness and the mechanical and thermomechanical characteristics of the films it produces are affected [49,50]. To facilitate charge carrier transfer, low molecular weight plasticizers have been added, resulting in a reduction in crystallinity, an increase in salt dissociation capacity and producing a jelly thin film, as seen in Figure 6a,b.

### 2.3. Complex Impedance Spectroscopy (CIS) and Ion Transport Study

**Figure 7:**
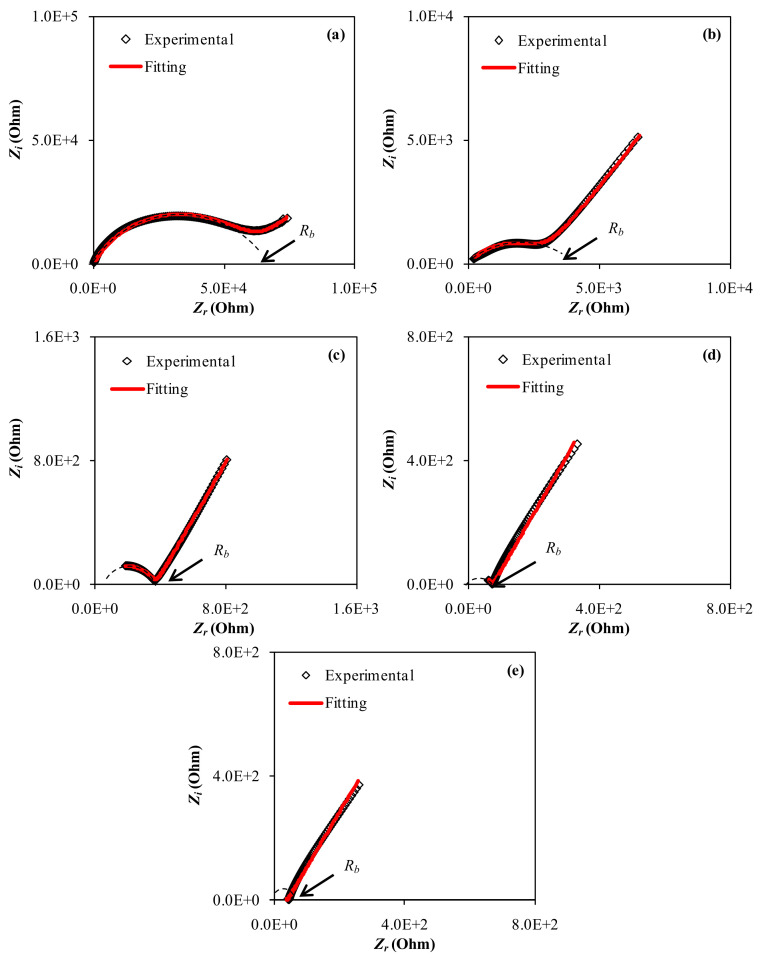
EIS plots for (**a**) CSPVNG11, (**b**) CSPVNG22, (**c**) CSPVNG33, (**d**) CSPVNG44 and (**e**) CSPVNG55 electrolyte films.

It is required to conduct non-destructive testing in order to distinguish a wide range of materials. As a tool for studying heterogeneous systems, dielectric impedance spectroscopy, which measures conductivity and permittivity as a function of frequency at different temperatures, may reveal their structure [51]. The CIS response is seen in the Nyquist plot of all the samples, as shown in Figure 7a–e. In the high frequency and low frequency regions, the responses take the shape of a semicircle and a tail (spike), respectively. On the one hand, at high frequencies, the incomplete semicircle is primarily associated with bulk resistance (a bulk property). The development of a spike, on the other hand, denotes the establishment of double layer capacitance at the electrode/sample interface at low frequencies [52].

The facility to recognize the relaxation frequency and to separate electrode (low frequency spike) and bulk (high frequency semicircular region) affects are two of the benefits of the CIS technique. Real (Z′) and imaginary (Z′′) components of impedance can be obtained using the CIS approach across a large frequency range. This technique has been effectively utilized to assess DC ionic conductivity and ionic conductor activation energy in recent years [44,53]. An electrochemical sample holder must be exposed to an alternating voltage across a wide frequency range for CIS measurements [54]. The direct correlation between the system’s response and the proposed equivalent circuit, which represents the system’s electrical behavior, is a key element of CIS [55,56]. In physics, a dielectric material’s dissipative response is characterized by a resistance (R), whereas its storing response is characterized by a capacitance (C) [56,57]. One of the outputs of electrical impedance spectroscopy is a graph of the imaginary component of the impedance vs. the real component, CIS. This graph can be used to derive information about the expected equivalent circuit. The impedance charts should have a straight line parallel to the imaginary axis at low frequencies; instead, the blocking electrode polarization effect (double layer capacitance) produces the curvature [58,59,60].

**Figure 8:**
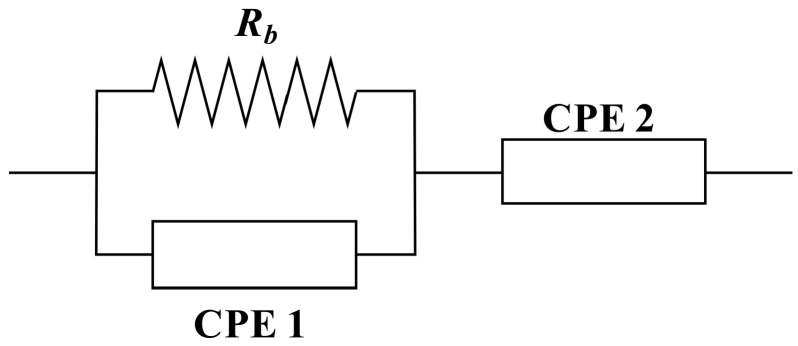
The equivalent electrical circuit (EEC).

It will be simple to specify the mechanism of the system after fitting experimental spectra and analyzing using the electrical equivalent circuit (EEC) technique [59]. Figure 7 and Figure 8 show the experimental impedance spectra, as well as the related EEC models for all of the electrolyte samples. In the Nyquist plots displayed in the insets of the bulk resistance (R_b_) for charge carriers in electrolyte systems, as well as two constant phase elements (CPEs); CPE1 and CPE2, can be seen Figure 8. The EEC is made up of a succession of R_b_ and CPE1 in parallel with a second CPE2 (from the tilted spike). The impedance’s ZCPE component is written as follows [55,61,62]:

(1) $$Z_{\mathrm{CPE}}=\frac{1}{{C\omega }^{p\;}}\left[\cos\left(\frac{\pi p}{2}\right)-i\;\sin\left(\frac{\pi p}{2}\right)\right]\;$$

where CPE capacitance and the angular frequency are symbolized as C and ω, and represents the departure of the plot from the vertical axis in complex impedance spectra. It is important to point out that CPE and capacitor are commonly used interchangeably in the context of an EEC modeling.

The overall mathematical expression of the equivalent circuit, and the real (Z_r_) and imaginary (Z_i_) complex impedance (Z*) values may be written as follows [55,61,62]:

(2) $$Z_{r}=\frac{N+R_{b}}{M+T}+\frac{\cos(\frac{{\pi p}_{2}}{2})}{C_{2}\omega^{p2}}\;$$

(3) $$Z_{i}=\frac{L}{M+T}+\frac{\sin(\frac{{\pi p}_{2}}{2})}{C_{2}\omega^{p2}}\;$$

where $N=R_{b}^{2}C_{1}\omega^{p1}\cos\left(\frac{{\pi p}_{1}}{2}\right)$, $M=2R_{b}C_{1}\omega^{p}\cos\left(\frac{\pi p}{2}\right)$, $L=R_{b}^{2}C_{1}\omega^{p1}\sin\left(\frac{{\pi p}_{1}}{2}\right)$ and $T=R_{b}^{2}C_{1}^{2}\omega^{2p}+1$.

The real and imaginary sections of semicircles are attributed to the first half of Equations (8) and (9), while spike lines are attributed to the second part. Where C_1_ indicates CPE1’s bulk capacitance and C_2_ denotes CPE2’s capacitance at the electrode-electrolyte interface. In Table 3, the EEC fitting parameters are listed. In the high frequency region, the semicircle size drops significantly as the amount of GL increases, as seen in the impedance spectra. Figure 7a,b show how to model an incomplete semicircle in which the value of R_b_ is in series with one CPE element and parallel with another CPE element (at low frequency tail). Figure 7c–e illustrate the complete elimination of the incomplete semicircle. Given the R_b_ values and the film thickness, calculation of the DC conductivity can be performed using Equation (4) [63].

(4) $$\sigma_{\mathrm{dc}}=\left(\frac{1}{R_{b}}\right)\times \left(\frac{t}{A}\right)\;$$

Table 4 shows the computed DC conductivity and bulk resistance (R_b_) values, as well as all the transport parameters of the plasticized PBE systems, including diffusion (D), mobility (µ) and charge carrier density (n). The quantity and sort of plasticizer have a big impact on the PEs’ characteristics. GL is the mainly often used and least expensive plasticizer [64]. After water, it is one of the most well-known polar compounds. It is completely water soluble, has a low toxicity compared to other organic solvents, and is completely biodegradable [27]. Furthermore, renewable resources are abundant on the earth and have a low market price. The relatively high conductivity can be explained by the massive ion movement, as demonstrated by the semicircle disappearing in the high frequency area of the spectra. As a result, GL is an effective plasticizer for reducing crystallinity and increasing ion mobility, which increases overall DC conductivity. According to Kaori Kobayashi et al., the addition of GL plasticizer reduced the glass transition temperature and increased the dc conductivity (σ_dc_) of poly (ethylene carbonate) (PEC): LiPF6Telectrolyte [65]. 

The heart of electrochemical devices is PEs. The majority of solid-state electrochemistry research is focused on the development of high ion-conducting materials for energy conversion and storage [66]. Massive research efforts over the past two decades have resulted in systems with enhanced conductivity and transport characteristics, and PEs fall into this category [66,67,68]. Transport parameters are crucial properties that should be considered in device applications. PEs with performance transport properties are the focus of many research groups.

Ion number density (n), diffusion coefficient (D), and mobility (μ) may be calculated from the impedance data of all systems by utilizing the following relations [69,70,71]:

(5) $$D=\left\{\frac{{\left(K_{2}\varepsilon_{0}\varepsilon_{r}A\right)}^{2}}{\tau_{2}}\right\}$$

where $\varepsilon_{r}$ and $\varepsilon_{0}$ signify the dielectric constant and the permittivity of the space, respectively. The reciprocal of ω is represented by $\tau_{2}$, which is matching to the smallest value in $Z_{i}$. From Equation (5), it is noticeable that three parameters influence the value of D, which are K_2_, ε_r_ and τ_2_. Thus, it is difficult to obtain D and µ values follow the trend of n value.

(6) $$\mu =\left\{\frac{\mathrm{eD}}{K_{b}T}\right\}\;$$

where ${\;k}_{b}$ and  T  refer to the Boltzmann constant and absolute temperature, respectively. DC conductivity ($\sigma_{\mathrm{dc}}$) and Ion number density (n) are presented by the following equations, respectively:

(7) $$\sigma_{dc}=ne\mu \;$$

(8) $$n=\left\{\frac{\sigma_{dc}K_{b}T\varepsilon \tau_{2}}{{\left(eK_{2}\varepsilon_{0}\varepsilon_{r}A\right)}^{2}}\right\}\;$$

From Table 4, is clear that the number density improved from $1.14\times 10^{15}\;{\mathrm{cm}}^{-3}$ to $4.24\times 10^{20}{\;\mathrm{cm}}^{-3}$. A large network of ion conduction may be formed by the incorporation of plasticizer molecules into the host polymer, as shown in Figure 6a [72]. According to recent research, the conductivity of thin films may be improved by increasing the amount of existing hydroxyl groups [73]. To lower intermolecular tensions, increase the mobility of its polymeric chains and enhance mechanical properties such as the extensibility of the resultant film, GL was essential [28,74].

### 2.4. Dielectric Properties

**Figure 9:**
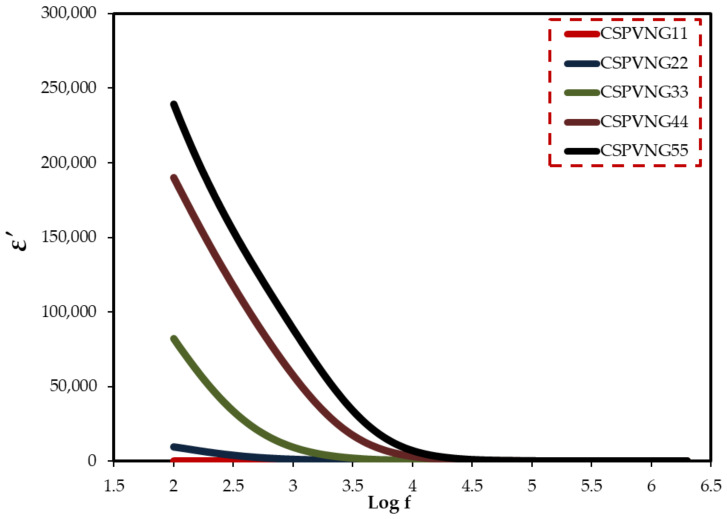
Dielectric constant versus log(f) for the electrolyte samples.

**Figure 10:**
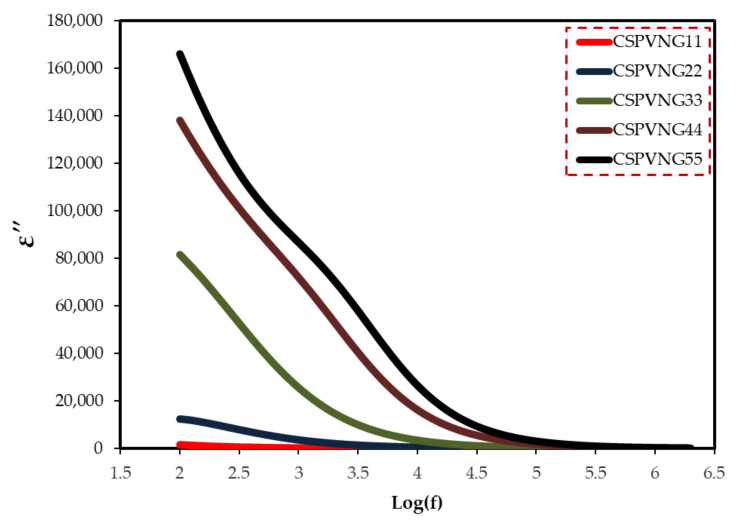
Dielectric loss versus log(f) for the electrolyte samples.

For determining the electrical characteristics of many materials, including glasses, semiconductors, polymers, and transition metal oxides, impedance spectroscopy may be a valuable instrument [9,40]. CIS relies on a number of other measured or calculated parameters. The measurement, analysis, and charting of some or all of the four impedance-related functions (Z*, Y*, M*, and ε*) in the complex plane is referred to as impedance spectroscopy [75]. Dielectric measurements (ε*), such as dielectric constant (ε′) and dielectric loss (ε′′), disclose a lot about polymer chemical and structural behavior. Using Equations (9) and (10), the real and imaginary parts of dielectric function were evaluated [76]. Because of the electrode polarization (EP) effect [77], both of them (ɛ′ and ɛ′′) are quite high at low frequencies, as seen in Figure 9 and Figure 10. The inclusion of another polymer or a dopant in the polymer has a significant impact on these properties (ɛ′ and ɛ′′) [56]. In polymer electrolyte systems the polarization can be resulted from both permanent dipole of host matrix functional groups and space charge polarization at the electrode/electrolyte interface. As can be seen in Figure 10, the dielectric loss has recorded high value at low frequency region due to the plenty of time that is provided by the applied field for re-orientation. In addition, the increasing plasticizer has improved the rotation freedom of the site groups to response quickly to the applied field. These two factors have caused an enhancement in dielectric loss value. However, at high frequency regions such a high value is not observed due to the fast switching of the applied field.

(9) $$\varepsilon^{\prime }=\frac{Z_{i}}{{\omega C}_{0}\left(Z_{r}^{2}+Z_{i}^{2}\right)}\;$$

(10) $$\varepsilon^{\prime \prime }=\frac{Z_{r}}{{\omega C}_{0}\left(Z_{r}^{2}+Z_{i}^{2}\right)}\;$$

The vacuum capacitance, $C_{o}$, is provided by the formula $\varepsilon_{o}A/t,$ where $\varepsilon_{o}$ is the free space permittivity, which is to $8.85\times 10^{-12}\;F/m$. If f is the applied field frequency, the angular frequency (ω) is equal to 2πf.

At low frequencies, ion migration and polarization have a significant impact on dielectric properties. For polymer/filler composites, this phenomenon is not possible. Due to the delayed dielectric relaxation of various polarizations in polymer composites, the permittivity falls gradually with increasing frequency. Permittivity has a low frequency dependency, which is advantageous across a large frequency range [78].

The migration of ions from one site to another in solid PEs will cause the electric potential of the environment to be perturbed. The perturb potential will have an impact on the movement of the other ions in this location. Non-exponential decay, or conduction processes with a dispersion of relaxation time, will occur as a result of such cooperative ion motion [79]. The behavior of bulk dielectric constant and bulk DC conductivity is essentially identical with GL content, as can be seen. This finding demonstrates that DC conductivity is greatly influenced by the dielectric constant. It is important to note that as the plasticizer concentration rises, the dielectric dispersion switches to the high frequency region. Both DC conductivity and ɛ′ are clearly lower for Gl content of 10 to 20 Gl. When crystallites cover a significant percentage of the polymer, the conductivity and dielectric constant are dramatically reduced, which is an undesirable circumstance [80]. Most of the dielectric constant is reduced by structural densification (i.e., the rise in crystallinity between the provided lamellae). Charge carrier mobility is hindered during the remainder of the amorphous phase as a consequence. The conduction channel may become unstable even if the amorphous phase composition changes slightly in such a compact arrangement. There is also the possibility that electrode contact loss and sample stiffness are to blame for such a large conductivity decline or resistivity rise [81]. Usually, the dominance of the EP effect can be attributed to significant charge carrier buildup at high GL concentration or at high temperature at the electrode/electrolyte interface because at these situations the semicircle disappears in CIS plots [82].

When an electric field is given to a solid substance, it causes ion conduction between favorable binding sites. The tail at low frequencies may be owing to the electrode/electrolyte interfaces blocking mobile ions and/or equipment constraints in viewing the low-frequency electrode polarization region [83]. The magnitude of ionic conductivity is described in theory as

(11) $$\sigma =\sum q_{i}n_{i}\mu_{i}\;$$

where $n_{i}$ denotes the number of carriers, and $q_{i}$ and $\mu_{i}$ denote the carriers’ charge and mobility, respectively. To achieve good ionic conductivity, you will need a lot of ion carriers with a large mobility. As a result, it is critical to discover materials that match these requirements [84]. The concentration of charge carriers’ growths when the dielectric constant rises $\left({\;n}_{i}=n_{o}\exp\left(-\frac{U}{{\;\varepsilon^{\prime }\;K}_{B}T}\right)\right)$, where U denotes the dissociation energy, resulting in anincrease in DC conductivity $\left(\sigma =\sum q_{i}n_{i}\mu_{i}\right)$.

### 2.5. Tanδ Study

**Figure 11:**
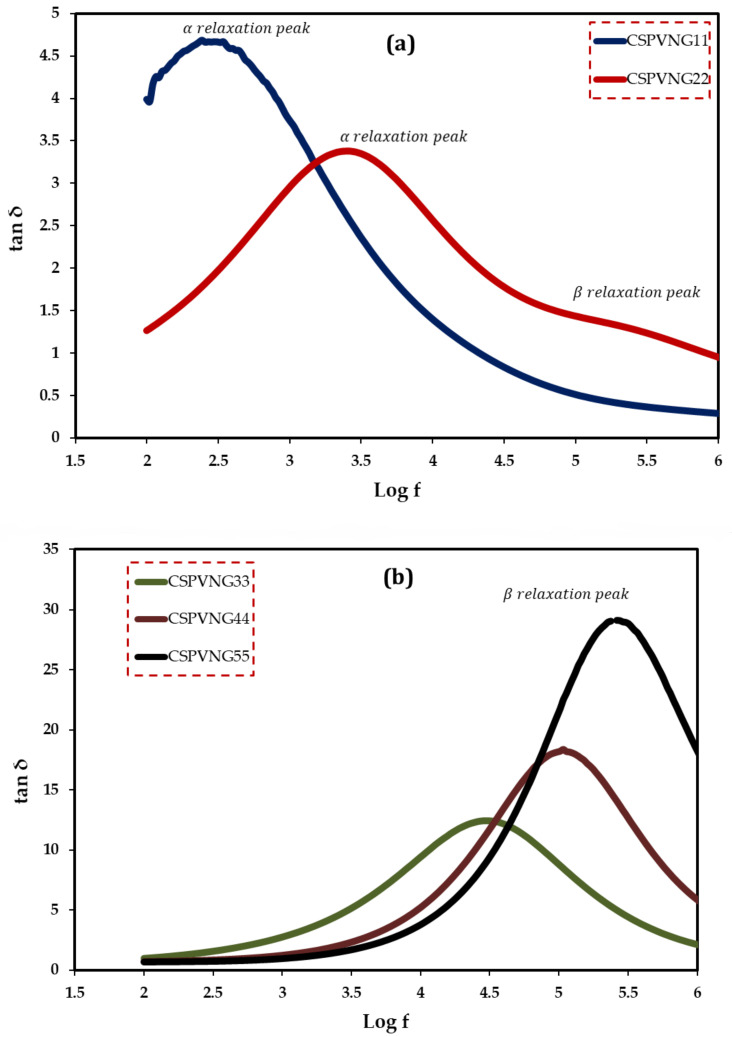
Loss tangent versus log (f) for (**a**) CSPVNG11 and CSPVNG12, and (**b**) CSPVNG33, CSPVNG44 and CSPVNG55  the electrolyte samples.

Broadband dielectric spectroscopy has been revealed to be a very efficient method for studying polymeric system relaxation processes. The complex dielectric utility, ɛ*, which consists of the dielectric constant and loss, is a material parameter that is affected by frequency, temperature, and structure, and so the tan, which is the ratio of ɛ“ to ɛ‘, is likewise frequency dependent. The plot of tanδ dielectric relaxation peaks as a function of frequency at room temperature for all samples is explored in this section. The aim of this study is to learn more about the relaxation processes and structure of polymeric-based materials. The study of dielectric relaxation can be a useful technique for understanding dipole relaxation in PEs. The dielectric relaxation processes are usually linked to one or more of the material’s polarization processes. The dipolar polarization and polarization owing to migrating charges are the two major components of polymer dielectric response. In frequencies less than 10^9^ Hz, dipolar and migrating charge polarizations can be identified [85]. To our understanding, there is no literature that discusses the use of loss tangent (Tanδ) relaxation peaks in determining polymeric structural attributes. The mechanism of ion transport is presently under debate among scientists. As a result, the study’s primary contribution is to provide the first experimental data and insights into the tan relaxation peaks and structure identification. The dielectric loss peak has been recognized as useful in investigating relaxations such as, α, β and γ, which are connected to dipole rotation in the crystalline phase, dipole orientation in amorphous areas, and side group or end-group movement in the amorphous phase, respectively [86]. α relaxation occurs at low frequencies most of the time, but β relaxation can be seen at intermediate to higher frequencies, and it transitions to higher frequencies as the amorphous phases increases [55]. The height of the peak associated with the amorphous phase at high frequency is clearly increased as the GL content increases. The results of the morphological and impedance analyses are exactly in line with these conclusions. As a result, the loss tangent peak can be used to determine structural features of materials in a sensitive manner. PEs are known to be heterogeneous materials, as both amorphous and crystalline phases exist. At high GL concentrations, the single peak disappears, implying system homogeneity [87]. The findings in this work suggest that tanδ relaxation peaks can be used to distinguish between crystalline and amorphous polymer phases. Furthermore, one peak formed at 10 wt.% of GL, while two loss peaks appeared at 20 wt.% of GL, as shown in Figure 11a. In both crystalline and amorphous regions, the peaks are connected to dipole orientation. The α -relaxation loss peak was discovered to be narrower and asymmetrical than the β -relaxation peak. The α -relaxation is partially filtered at low frequencies due to ionic conductivity [55,88]. While Figure 11b shows that α -relaxation loss peak has been disappeared, and β -relaxation peak has dominated due to the transfer of these systems from crystalline phase to fully amorphous phase, which is supported by XRD results (Figure 4).

### 2.6. Electric Modulus and Relaxation Study

**Figure 12:**
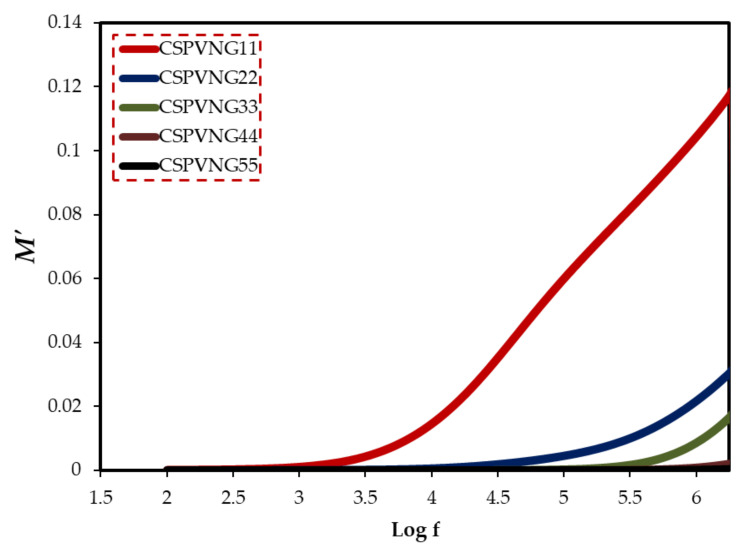
The real part of the electric modulus versus log (f) for the electrolyte samples.

**Figure 13:**
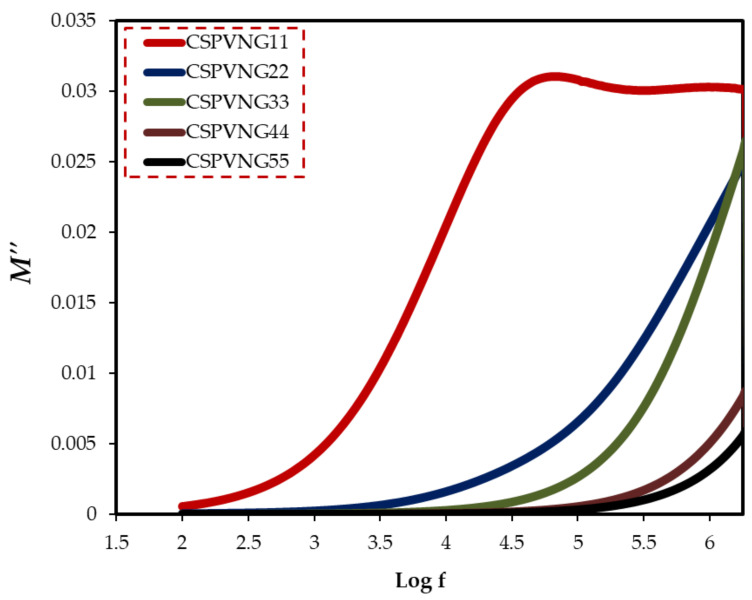
The imaginary part of the electric modulus versus log (f) for all electrolyte samples.

The material is exposed to an alternating electric field, which is generated by applying a sinusoidal voltage, in dielectric measurements; this process causes dipoles in the material to align, resulting in polarization. Dipoles on the side chain of the polymer backbone can cause dipolar polarization in polymeric materials, as well as the presence of ion translational diffusion [89]. Because of the huge conductivity effects, dielectric parameters should be expressed in terms of the complex electric modulus (M* = 1/ε*) [90]. It can be deduced from the electric modulus study that ion transport happens either by polymer segmental relaxation or conductivity relaxation [91]. Electrical relaxation phenomena in PE are known to be caused by phase transitions, interfacial effects, and polarization or conductivity mechanisms [92]. Macedo et al. devised the electric modulus formalism to limit the effect of electrode polarization. Equations (12) and (13) were used to compute the real and imaginary components of electric modulus [93]. The peak maximum in the imaginary part of the electric modulus indicates that the sample is an ion conductor. Figure 12 depicts the frequency dependence of Mʹ for various GL plasticizer concentrations. Figure 13 shows that a distinct relaxation peak can be detected in the imaginary part of modulus (M″) spectra, which is associated with conductivity processes, however no peak can be found in the dielectric loss spectra. This indicates that ionic and polymer segmental movements are highly connected, as evidenced by a single peak in the M” spectra and no equivalent characteristic in dielectric loss spectra (Figure 13) [94]. As a result, charge migration of ions between coordinated sites of the polymer and segmental relaxation of the polymer are both involved in conduction in PEs.

(12) $$\;M^{\prime }={\omega C}_{0}Z_{i}\;$$

(13) $$M^{\prime \prime }={\omega C}_{0}Z_{r}\;$$

**Figure 14:**
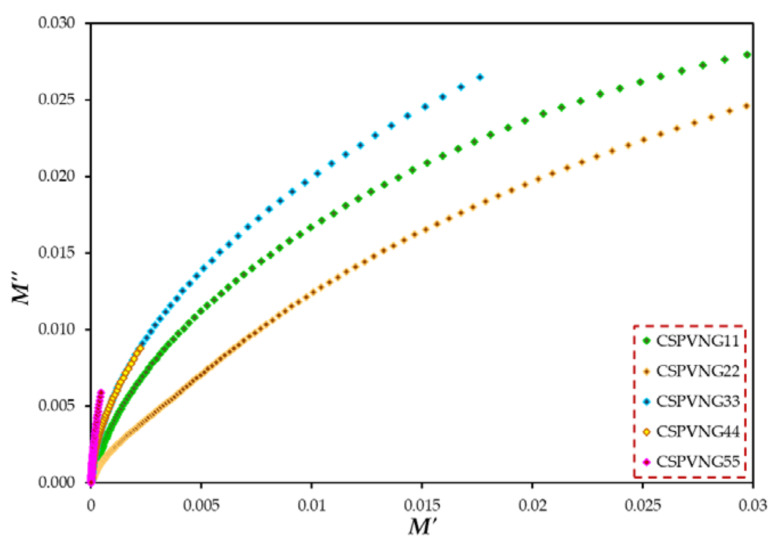
M′′-M′ plot for all plasticized SPE films.

The use of Argand (M′′ vs. M′) plots to decide whether a relaxation dynamic fits in to viscoelastic or conductivity relaxation is a well-known approach. The type of relaxation processes in the current PEs can be revealed. The Argand curves for all of the samples are shown in Figure 14 at room temperature. According to [95], if the Argand plot between the imaginary component (M′′) and the real part (M′) of the electric modulus displays semicircular behavior, the relaxation is due to conductivity relaxation; otherwise, viscoelastic relaxation (or polymer molecular relaxation) is responsible [96]. The circle diameters do not line up with the real axis. This means that ion transport happens in all samples via viscoelastic relaxation. The conductivity relaxation differs significantly from the viscoelastic relaxations found in polymers. The conductivity relaxation corresponds to a single-relaxation-time Debye model process, whereas viscoelastic relaxations are known to have a dispersion of relaxation times [97].

### 2.7. AC Conductivity Analysis (σ_ac_)

Even though experiments have been carried out on a broad range of semiconducting and insulating materials, the literature on AC conductivity in disorder solids and its characteristics with composition is valuable. In general, frequency-dependent conductivity follows a power-law behavior (σ~ω^s^) [98]. The properties of crystalline semiconductors and insulators have been determined by several solid state theories, but when these theories are applied to data on polymers, the predictions they produce are not sufficiently unique, making it surprisingly difficult to determine all the important characteristics of electrical conduction in polymers even today [99]. The σ_ac_ were evaluated from the real (Z_r_) and imaginary (Z_i_) parts of complex impedance (Z*) using Equation (14)_._

(14) $$\sigma_{\mathrm{ac}}^{\prime }=\left[\frac{Z_{r}}{Z_{r}^{2}+Z_{i}^{2}}\right]\times \left(\frac{t}{A}\right)\;$$

**Figure 15:**
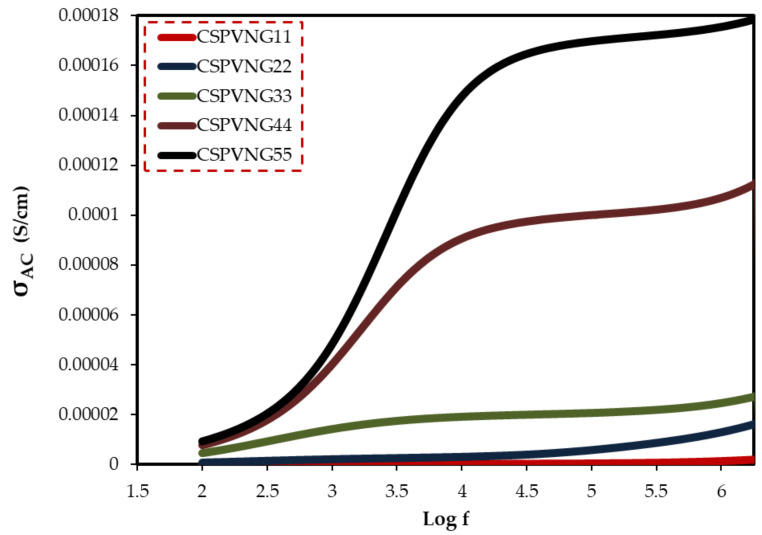
AC conductivity spectra.

Figure 15 shows the AC conductivity spectra of plasticized PVA:CS:NaBr sheets at ambient temperatures. At high frequencies, ac conductivity in disordered solids is highly dispersed, making it one of the most distinctive properties of electrical conduction in these materials. AC methods are widely used to study electrical properties of ionically conducting materials, which would need the construction of non-blocking electrodes for DC research [100]. Frequency dependent data may be used to highlight the contributions of bulk materials (high frequency semicircle area), grain boundaries, and electrode-electrolyte effects (low frequency spike region) [101]. In the GL range of 11 to 33 wt.%, the ac conductivity spectra (see Figure 15) may be separated into three discrete regions. The electrode-electrolyte interfacial phenomenon known as electrode polarization (EP) effect caused the low frequency area (region I) to appear as a spike [102]. It is worth noting that a linear relation between the spike region and GL content can be observed. A plateau of ac conductivity is identified in the intermediate frequency band (region II), which correlates to DC conductivity. As a result of increased electrode polarization (EP) and a shift to the higher frequency side, this region (plateau region) grows dramatically with increasing GL content. At high GL concentrations, the disappearance of the high frequency semicircle in the impedance plot (Z_i_-Z_r_) is closely related to the lowering of dispersion region (Figure 7a–e). These dispersion areas are critical for determining the ion conduction type in solid PEs.

The Jonscher’s relation is a good way to describe the link between ac conductivity and charge carrier motion [103]:

(15) $$\sigma_{dc}\left(\omega \right)=\sigma_{dc}+A\omega^{s}\left(0<s<1\right)$$

where *σ_dc_* denotes the frequency-independent dc conductivity, A denotes a temperature-dependent constant, and s  denotes the charge carrier interactions during hopping processes [104]. The second component is caused by accumulated interfacial charges and permanent/induced polarization (restricted mobility) of the dipoles. As frequency rises, the total conductivity of the second component due to polarization increases, according to Equation (15) [105]. *σ’_ac_*(ω) represents the charge transport mechanism and many-body interactions among charge carriers [104,106]. 

## 3. Conclusions

Finally, the casting approach was employed to make plasticized PBEs based on polyvinyl alcohol (PVA): chitosan (CS) polymers. The samples structural and electrical properties were investigated using a variety of techniques including XRD, FTIR and EIS. There is interaction at the molecular level, as shown by shifting peaks and changes in the intensity of FTIR bands. PVA and CS polymers behave semicrystallinly, as shown by the XRD data. It was determined that the samples inserted with high content of GL were amorphous due to the XRD pattern exhibiting a wide hum. The GL plasticizer fastened the dissociation of NaBr salt and reduced the crystallinity of polymers. For a sample containing 55 wt% GL, the CIS approach revealed a maximum ionic conductivity of $3.8\times 10^{-4}$ S/cm. Each electrolyte’s CIS data were fitted with EECs to have a comprehensive understanding of the ion-conducting systems’ full electrical properties. The DC conductivity and carrier density increased with increasing GL ratio. With the help of EEC modeling, the transport parameters associated with dissociated ions are calculated. The conductivity measurement was connected to the dielectric characteristics. High dielectric constant and dielectric loss were recorded at low frequencies. The relaxation behaviors of the samples are examined using loss tangent and electric modulus graphs. The peak detected in the spectra of tanδ and M” plots and the distribution of data points are asymmetric beside the peaks. This confirms that the movement of ions and their contribution to conductivity belongs to the viscoelastic relaxation type. The high ionic conductivity and improved transport properties (n, μ and D) revealed the suitableness of the films for energy storage device applications. 

## 4. Materials and Methods

### 4.1. Material Components

PVA (MW 89,000–98,000 g/mol, 99+% hydrolyzed) and chitosan (CS) (MW 310,000–375,000 g/mol, Sigma-Aldrich, St. Louis, MO, USA) are the polymeric materials. Other raw materials employed included sodium bromide (NaBr) (MW 102.89 g/mol, Sigma-Aldrich) salt as a dopant, acetic acid (CH3COOH) solution and distill water as a solvent, and GL (C3H8O3) (MW 92.09 g/mol, Sigma-Aldrich) as a plasticizer. No additional purification was necessary.

### 4.2. Electrolyte Preparation Using Polymer Blends

**Figure 16:**
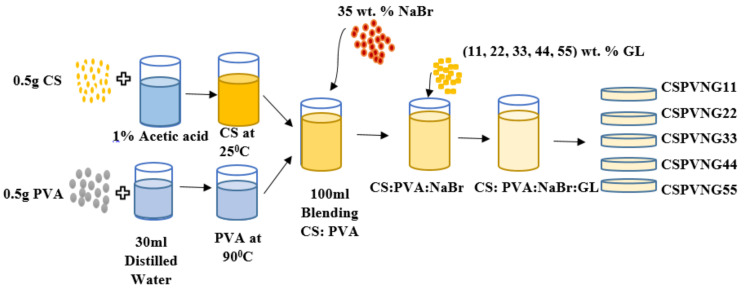
Schematic processes for the fabrication of the plasticized PE samples.

Acetic acid solvents were prepared by adding 1 mL of glacial acetic acid into 99 mL of distilled water. 0.5 g of CS was dissolved in 70 mL of the prepared (1%) acetic acid at ambient temperature and 0.5 g of PVA was dissolved in 30 mL distilled water at 90 °C under stirring. After cooling to room temperature, the PVA solution was mixed with dissolved CS. Using a magnetic stirrer, the solution was continuously stirred for 24 h to obtain an identical PVA:CS solution. To make ion-conducting PEs, sodium salt was used. For this purpose, 35 wt.% NaBr salt was added to the PVA:CS solutions under stirring and then addition of varying concentrations (11, 22, 33, 44, and 55 wt.%) of GL as plasticizers into the PVA:CS:NaBr solution were carried out. In order to obtain a dry film free of solvent at room temperature, the solutions were deposited in numerous plastic Petri dishes (8 cm in diameter). This procedure is seen in the Figure 16. The 0.023 mm-thick flexible films were then created. 

It was determined that CSPVNG0, CSPVNG11, CSPVNG22, CSPVNG33, CSPVNG44, and CSPVNG55 were the appropriate coding for the PVA:CS:NaBr mix systems doped with varied concentrations of GL. Table 5 depicts the film’s chemical composition.

### 4.3. XRD and FTIR Spectra Analysis

Bruker D8 ADVANCE X-ray powder diffractometer (Bruker, Berlin, Germany) with CuK*a* radiation sources (k = 1.54 A°) is used to record XRD patterns of pure and composition polymer films in the region of 10–80° in order to provide information about their crystal structure. The interaction between the composite components was studied using a Perkin Elmer FTIR spectrometer with a range of 500 to 4000 cm^−1^ and a resolution of 2 cm^−1^.

### 4.4. Electrical Impedance Spectroscopy (EIS)

**Figure 17:**
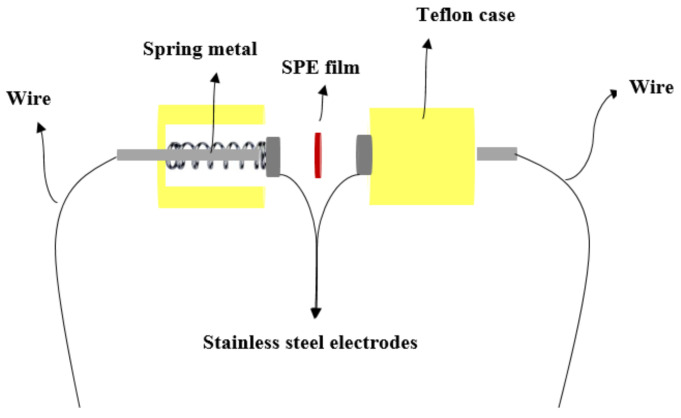
Schematic the stainless-steel electrodes.

Complex impedance spectroscopy may be used to determine the electrical properties of materials and their interaction with electrically conducting electrodes. SPE films were cut into 2 cm diameter compact discs and placed between two stainless steel electrodes under spring pressure as shown in Figure 17.

HIOKI 3531 Z  Hi-tester, linked to a computer, was used to measure the impedance of the films throughout a frequency range of 100 Hz to 2 MHz. $Z_{r}$ and $Z_{i}$ are calculated by the software and controlled by it throughout the measuring process. An intersection of the plot and real impedance axis yielded bulk resistance ($R_{b}$), and the $Z_{r}$ and $Z_{i}$ values were presented as a Nyquist plot. The equation below may be used to determine conductivity [107,108].

In Equation (1), there are three parameters to consider: film thickness (t), bulk resistance ($R_{b}$), and active area (A). It is also possible to determine the real and imaginary components of dielectric and electric modulus using $Z_{r}$ and $Z_{i}$ by the Equations (9) and (10), (12) and (13), which are based on complex impedance (Z*) [107,108].

## Figures and Tables

**Table 1:** Purified (PVA), pure (CS) and blended pure (PVA:CS) FTIR data are shown.

Vibrational Modes	Pure PVA	Vibrational Modes	PVA:CS	Vibrational Modes	Pure CS
O–H stretching	3259.34	O–H stretching	3261.67	O–H stretching	3261.42
C–H stretching	2937.98	C–H stretching	2906.38	C–H stretching	2872.47
C=O	1710	C=O	1646.29	C=O	1636.33
$\left(C\text{–}H\right)\text{–}\mathrm{CH}2$	1653.51	N–H	1557.41	N–H	1540.59
OH–C–OH	1417.33	CH·OH	1405.89	CH·OH	1403.24
–C–O–C	1324.90	CH2–OH	1376.61	CH2–OH	1375.46
$\left(C\text{–}O\right)\text{–}C\text{–}\mathrm{OH}$	1084.88	$\left(C\text{–}O\right)\text{–}C\text{–}\mathrm{OH}$	1027.48	$\left(C\text{–}O\right)\text{–}C\text{–}\mathrm{OH}$	1022.98
C–C	834.16	C–C	834.08	C–C	–

**Table 2:** Assignments of FTIR bands for PVA:CS: NaBr:GL solid PEs.

Samples	Wavenumbers (cm^−1^)			
	C–O Band	NH_2_	C–H Band	O–H Band
CSPVNG0	1015.61	1545.66	-	-
CSPVNG11	1020.04	1558.79	-	3374.36
CSPVNG22	1039.06	1559.40	2938.77	3374.69
CSPVNG33	1032.10	1559.33	2938.70	3317.19
CSPVNG44	1031.21	1559.41	2937.25	3316.19
CSPVNG55	1031.29	1559.47	2936.4	3312.77

**Table 3:** Circuit elements of the plasticized PBE systems.

Sample	$p_{1}\;(rad)$	$p_{2}\;(rad)$	CPE1 (F)	CPE2 (F)
CSPVNG11	0.71	0.52	$6.67\times 10^{-9}$	$1.54\times 10^{-6}$
CSPVNG22	0.60	0.60	$1.43\times 10^{-7}$	$3.33\times 10^{-6}$
CSPVNG33	0.73	0.68	$2.22\times 10^{-8}$	$1.35\times 10^{-5}$
CSPVNG44	0.71	0.68	$5.00\times 10^{-8}$	$2.38\times 10^{-5}$
CSPVNG55	0.64	0.67	$1.00\times 10^{-7}$	$2.94\times 10^{-5}$

**Table 4:** Transport parameters of the plasticized PBE systems.

Sample	Rb (Ω)	**σ** $\left(S\;cm^{-1}\right)$	$D\;(cm^{2}s^{-1})$	$\mu \;(cm^{2}V^{-1}s)$	$n\;(cm^{-3})$
CSPVNG11	62,600	$2.46\times 10^{-7}$	$3.48\times 10^{-5}$	$1.35\times 10^{-3}$	$1.14\times 10^{15}$
CSPVNG22	2815	$5.48\times 10^{-6}$	$1.52\times 10^{-5}$	$5.90\times 10^{-4}$	$5.79\times 10^{16}$
CSPVNG33	369	$4.18\times 10^{-5}$	$8.60\times 10^{-7}$	$3.35\times 10^{-5}$	$7.79\times 10^{18}$
CSPVNG44	73	$2.11\times 10^{-4}$	$4.06\times 10^{-7}$	$1.58\times 10^{-5}$	$8.34\times 10^{19}$
CSPVNG55	40	$3.86\times 10^{-4}$	$1.46\times 10^{-7}$	$5.67\times 10^{-6}$	$4.24\times 10^{20}$

**Table 5:** Solid PE based on PVA:CS:NaBr:GL.

Designation	CS:PVA:NaBr (g)	Glycerol Wt.%	Glycerol (g)
CSPVNG0	$\left(0.5:0.5:0.538\right)$	0	0
CSPVNG11	$\left(0.5:0.5:0.538\right)$	11	0.190
CSPVNG22	$\left(0.5:0.5:0.538\right)$	11	0.4339
CSPVNG33	$\left(0.5:0.5:0.538\right)$	22	0.758
CSPVNG44	$\left(0.5:0.5:0.538\right)$	33	1.209
CSPVNG55	$\left(0.5:0.5:0.538\right)$	44	1.880
